# A New Family of *q*-Supercongruences Modulo the Fourth Power of a Cyclotomic Polynomial

**DOI:** 10.1007/s00025-020-01272-7

**Published:** 2020-09-29

**Authors:** Victor J. W. Guo, Michael J. Schlosser

**Affiliations:** 1 School of Mathematics and Statistics, Huaiyin Normal University, Huai’an, 223300 Jiangsu People’s Republic of China; 2 Fakultät für Mathematik, Universität Wien, Oskar-Morgenstern-Platz 1, 1090 Vienna, Austria

**Keywords:** Primary 33D15, Secondary 11A07, 11B65, Basic hypergeometric series, supercongruences, *q*-congruences, cyclotomic polynomial, *q*-microscoping, Chinese remainder theorem for polynomials

## Abstract

We establish a new family of *q*-supercongruences modulo the fourth power of a cyclotomic polynomial, and give several related results. Our main ingredients are *q*-microscoping and the Chinese remainder theorem for polynomials.

## Introduction

More than one hundred years ago, Ramanujan mysteriously recorded a list of rapidly convergent series of $1/\pi$ (see [1, p. 352]), including

1.1 $$\begin{aligned} \sum _{n=0}^\infty \frac{\left( {\begin{array}{c}4n\\ 2n\end{array}}\right) {\left( {\begin{array}{c}2n\\ n\end{array}}\right) }^2}{2^{8n}3^{2n}}(8n+1) =\frac{2\sqrt{3}}{\pi }, \end{aligned}$$

which he later published in [19, Equation (40)]. In 1997, Van Hamme [23] observed that 13 Ramanujan’s and Ramanujan-type formulas possess interesting *p*-adic analogues, such as

1.2 $$\begin{aligned} \sum _{k=0}^{(p-1)/2} (4k+1)\frac{(\frac{1}{2})_k^4}{k!^4}&\equiv p\pmod {p^3}, \end{aligned}$$

where $p>3$ is a prime and $(a)_n=a(a+1)\cdots (a+n-1)$ is the Pochhammer symbol. Van Hamme [23, (C.2)] himself proved (1.2) and two of the other supercongruences of his list. The supercongruence (1.2) was later proved to be true modulo $p^4$ by Long [17]. For more Ramanujan-type supercongruences, we refer the reader to Zudilin’s paper [26].

During the past few years, *q*-analogues of congruences and supercongruences have been investigated by many authors (see [3–16, 18, 21, 22, 24, 25, 27]). For instance, using a method similar to that used in [26], the first author and Wang [12, Theorem 1.2] gave a *q*-analogue of (1.2): for odd *n*,

1.3 $$\begin{aligned}&\sum _{k=0}^{(n-1)/2}[4k+1]\frac{(q;q^2)_k^4}{(q^2;q^2)_k^4}\nonumber \\&\quad \equiv q^{(1-n)/2}[n]+\frac{(n^2-1)(1-q)^2}{24}q^{(1-n)/2}[n]^3 \pmod {[n]\Phi _n(q)^3}. \end{aligned}$$

Moreover, the first author and Zudilin [13] devised a method of ‘creative microscoping’ to prove that, for any positive integer *n* with $\gcd (n,6)=1$,

1.4 $$\begin{aligned}&\sum _{k=0}^{(n-1)/2}[8k+1]\frac{(q;q^2)_k^2 (q;q^2)_{2k}}{(q^2;q^2)_{2k}(q^6;q^6)_k^2}q^{2k^2}\nonumber \\&\quad \equiv q^{-(n-1)/2}[n]\biggl (\frac{-3}{n}\biggr ) \pmod {[n]\Phi _n(q)^2}, \end{aligned}$$

where $(\frac{-3}{\cdot })$ is the Jacobi symbol, see [13, Theorem 1.1, Equation (6)]. Here it is appropriate to recall the standard *q*-hypergeometric notation: $(a;q)_n=(1-a)(1-aq)\cdots (1-aq^{n-1})$ is the *q-shifted factorial*, with the condensed notation $(a_1,\dots ,a_m;q)_n=(a_1;q)_n\cdots (a_m;q)_n$ for products of *q*-shifted factorials; $[n]=[n]_q=(1-q^n)/(1-q)$ is the *q-integer*; and $\Phi _n(q)$ stands for the *n*th *cyclotomic polynomial* in *q*:

$$\begin{aligned} \Phi _n(q)=\prod _{\begin{array}{c} 1\leqslant k\leqslant n\\ \gcd (k,n)=1 \end{array}}(q-\zeta _n^k), \end{aligned}$$

where $\zeta _n$ denotes an *n*th primitive root of unity.

Clearly, the *q*-supercongruence (1.4) is a *q*-analogue of the following result (see [20, Conjecture 5.6]):

1.5 $$\begin{aligned} \sum _{k=0}^{(p-1)/2}(8k+1)\frac{\left( {\begin{array}{c}4k\\ 2k\end{array}}\right) {\left( {\begin{array}{c}2k\\ k\end{array}}\right) }^2}{2^{8k}3^{2k}} \equiv p\biggl (\frac{-3}{p}\biggr )\pmod {p^3} \quad \text {for }p>3\hbox { prime}, \end{aligned}$$

which is a *p*-adic analogue of (1.1). This means that by letting $q\rightarrow 1$ in (1.4) one obtains (1.5). We point out that no other proofs of (1.5) are known up to now.

The first author and Zudilin [13, Theorem 4.2] also gave a two-parameter generalization of (1.3) as follows: for odd *n*, modulo $[n](1-aq^n)(a-q^n)$,

$$\begin{aligned} \sum _{k=0}^{m}[4k+1]\frac{(aq,q/a,q/b,q;q^2)_k}{(aq^2,q^2/a,bq^2,q^2;q^2)_k}b^k \equiv \frac{(b/q)^{(n-1)/2} (q^2/b;q^2)_{(n-1)/2}}{(bq^2;q^2)_{(n-1)/2}}[n], \end{aligned}$$

where $m=(n-1)/2$ or $(n-1)$. Recently, based on the above *q*-congruence, by applying the Chinese remainder theorem for coprime polynomials, the first author [6, Theorem 1.1] succeeded in giving a full parametric generalization of (1.3): modulo $[n]\Phi _n(q)(1-aq^n)(a-q^n)$,

1.6 $$\begin{aligned}&\sum _{k=0}^{m}[4k+1]\frac{(aq,q/a,q,q;q^2)_k}{(aq^2,q^2/a,q^2,q^2;q^2)_k} \nonumber \\&\quad \equiv q^{(1-n)/2}[n]+q^{(1-n)/2}[n]\frac{(1-aq^n)(a-q^n)}{(1-a)^2}\left( 1-\frac{n(1-a)a^{(n-1)/2}}{1-a^n}\right) , \end{aligned}$$

where $m=(n-1)/2$ or $(n-1)$. Moreover, the present authors [8, Theorem 1.1, Equation (2a)] showed that, for odd $n>1$,

1.7 $$\begin{aligned} \sum _{k=0}^{(n+1)/2}[4k-1]\frac{(q^{-1};q^2)_k^4}{(q^2;q^2)_k^4} q^{4k} \equiv -(1+3q+q^2)[n]^4 \pmod {[n]^4\Phi _n(q)}. \end{aligned}$$

The main purpose of this paper is to establish a new family of *q*-supercongruences modulo the fourth power of a cyclotomic polynomial, which may somewhat be deemed a generalization of (1.3) and (1.7) modulo $[n]\Phi _n(q)^3$.

### Theorem 1.1

Let *d*, *n*, *r* be integers satisfying $d\geqslant 2$, $r\leqslant d-2$ (in particular, *r* may be negative), and $n\geqslant d-r$, such that *d* and *r* are coprime, and $n\equiv -r\pmod {d}$. Then

1.8 $$\begin{aligned}&\sum _{k=0}^{M}[2dk+r]\frac{(q^r;q^d)_k^4}{(q^d;q^d)_k^4}q^{(d-2r)k} \nonumber \\&\quad \equiv {\left\{ \begin{array}{ll} 0 \pmod {[n]\Phi _n(q)^3} &{}\text {if }d=2,\\ q^{r(n+r-dn)/d}\dfrac{(q^{2r};q^d)_{(dn-n-r)/d}}{(q^d;q^d)_{(dn-n-r)/d}}[dn-n] \pmod {[n]\Phi _n(q)^3} &{}\text {if }d\geqslant 3, \end{array}\right. } \end{aligned}$$

where $$M=(dn-n-r)/d$$ or $$n-1$$.

The proof is given in Sect. 3.

It is easy to see that the *q*-factorial $(q^{2r};q^d)_{(dn-n-r)/d}$ in (1.8) contains the factor $1-q^{(d-2)n}$ for $d\geqslant 3$. Since $1-q^{(d-2)n}\equiv [dn-n]\equiv 0\pmod {\Phi _n(q)}$ and $(q^d;q^d)_{(dn-n-r)/d}$ is coprime with $\Phi _n(q)$, we conclude from Theorem 1.1 that

$$\begin{aligned} \sum _{k=0}^{n-1}[2dk+r]\frac{(q^r;q^d)_k^4}{(q^d;q^d)_k^4}q^{(d-2r)k} \equiv 0\pmod {\Phi _n(q)^2} \end{aligned}$$

for $d\geqslant 3$. Our proof of Theorem 1.1 implies that the above *q*-congruence is further true modulo $[n]\Phi _n(q)$. Or the reader may check that the denominator of the reduced form of the fraction $(q^{2r};q^d)_{(dn-n-r)/d}/(q^d;q^d)_{(dn-n-r)/d}$ is coprime with [*n*].

We should point out that the present authors [10] have given a different generalization of (1.3) and (1.7) modulo $\Phi _n(q)^4$ as follows: for $d\geqslant 3$ and *n*, *r* satisfying the same condition as in Theorem 1.1, we have

1.9 $$\begin{aligned} \sum _{k=0}^{n-1}[2dk+r] \frac{(q^r;q^d)_k^{2d}}{(q^d;q^d)_k^{2d}}q^{d(d-1-r)k} \equiv 0 \pmod {\Phi _{n}(q)^4}. \end{aligned}$$

For $d\geqslant 3$ and $r=\pm 1$, Theorem 1.1 can be stated as follows:

### Corollary 1.2

Let *d* and *n* be positive integers with $d\geqslant 3$ and $n\equiv -1\pmod {d}$. Then

$$\begin{aligned}&\sum _{k=0}^{M}[2dk+1]\frac{(q;q^d)_k^4}{(q^d;q^d)_k^4}q^{(d-2)k}\\&\quad \equiv q^{(n-dn+1)/d}\frac{(q^2;q^d)_{(dn-n-1)/d}}{(q^d;q^d)_{(dn-n-1)/d}}[dn-n] \pmod {[n]\Phi _n(q)^3}, \end{aligned}$$

where $$M=(dn-n-1)/d$$ or $$n-1$$.

### Corollary 1.3

Let *d* and *n* be positive integers with $d\geqslant 3$ and $n\equiv 1\pmod {d}$. Then

$$\begin{aligned}&\sum _{k=0}^{M}[2dk-1]\frac{(q^{-1};q^d)_k^4}{(q^d;q^d)_k^4}q^{(d+2)k}\\&\quad \equiv q^{(dn-n+1)/d}\frac{(q^{-2};q^d)_{(dn-n+1)/d}}{(q^d;q^d)_{(dn-n+1)/d}}[dn-n] \pmod {[n]\Phi _n(q)^3}, \end{aligned}$$

where $$M=(dn-n+1)/d$$ or $$n-1$$.

We shall also prove the following *q*-congruence, which was originally conjectured by the first author and Zudilin [13, Conjecture 5.2].

### Theorem 1.4

Let *d* and *n* be positive integers with $d\geqslant 3$ and $n\equiv -1\pmod {d}$. Then

1.10 $$\begin{aligned} \sum _{k=0}^{n-1}[2dk+1]\frac{(aq,q/a;q^d)_k (q;q^d)_k^2}{(aq^d,q^d/a;q^d)_k (q^d;q^d)_k^2}q^{(d-2)k}&\equiv 0\pmod {[n]\Phi _n(q)}. \end{aligned}$$

Note that the $a=1$ case of (1.10) modulo $\Phi _n(q)^2$ has already been proved by the present authors [11, Theorem 2.4].

## Some Lemmas

We first give the following result which is a generalization of [11, Lemma 3.1], [10, Lemma 3] and [24, (2.1)].

### Lemma 2.1

Let *d*, *m* and *n* be positive integers with $m\leqslant n-1$. Let *r* be an integer satisfying $dm\equiv -r\pmod {n}$. Then, for $0\leqslant k\leqslant m$, we have

$$\begin{aligned} \frac{(aq^r;q^d)_{m-k}}{(q^d/a;q^d)_{m-k}} \equiv (-a)^{m-2k}\frac{(aq^r;q^d)_k}{(q^d/a;q^d)_k} q^{m(dm-d+2r)/2+(d-r)k} \pmod {\Phi _n(q)}. \end{aligned}$$

### Proof

In view of $q^{dm+r}\equiv q^n\equiv 1\pmod {\Phi _n(q)}$, we have

2.1 $$\begin{aligned} \frac{(aq^r;q^d)_{m} }{(q^d/a;q^d)_{m}}&=\frac{(1-aq^r)(1-aq^{d+r})\cdots (1-aq^{dm-d+r})}{(1-q^d/a)(1-q^{2d}/a)\cdots (1-q^{dm}/a)} \nonumber \\&\equiv \frac{(1-aq^r)(1-aq^{d+r})\cdots (1-aq^{dm-d+r})}{(1-q^{d-dm-r}/a)(1-q^{2d-dm-r}/a)\cdots (1-q^{-r}/a)}\nonumber \\&=(-a)^{m}q^{m(dm-d+2r)/2} \pmod {\Phi _n(q)}. \end{aligned}$$

Furthermore, modulo $\Phi _n(q)$, we get

$$\begin{aligned}&\frac{(aq^r;q^d)_{m-k}}{(q^d/a;q^d)_{m-k}}\\&\quad =\frac{(aq^r;q^d)_{m}}{(q^d/a;q^d)_{m}} \frac{(1-q^{dm-dk+d}/a)(1-q^{dm-dk+2d}/a)\cdots (1-q^{dm}/a)}{(1-aq^{dm-dk+r})(1-aq^{dm-dk+d+r})\cdots (1-aq^{dm-d+r})} \\&\quad \equiv \frac{(aq^r;q^d)_{m}}{(q^d/a;q^d)_{m}} \frac{(1-q^{d-dk-r}/a)(1-q^{2d-dk-r}/a)\cdots (1-q^{-r}/a)}{(1-aq^{-dk})(1-aq^{d-dk})\cdots (1-aq^{-d})} \\&\quad =\frac{(aq^r;q^d)_{m}}{(q^d/a;q^d)_{m}} \frac{(aq^r;q^d)_k}{(q^d/a;q^d)_k} a^{-2k} q^{(d-r)k}, \end{aligned}$$

which together with (2.1) establishes the assertion. $\square$

### Lemma 2.2

Let *d*, *n* be positive integers with $\gcd (d,n)=1$. Let *r* be an integer and let *a*, *b* be indeterminates. Then

2.2 $$\begin{aligned} \sum _{k=0}^{m}[2dk+r]\frac{(aq^r,q^r/a,q^r/b,q^r;q^d)_k}{(aq^d,q^d/a,bq^d,q^d;q^d)_k} b^k q^{(d-2r)k}&\equiv 0\pmod {[n]}, \end{aligned}$$

2.3 $$\begin{aligned} \sum _{k=0}^{n-1}[2dk+r]\frac{(aq^r,q^r/a,q^r/b,q^r;q^d)_k}{(aq^d,q^d/a,bq^d,q^d;q^d)_k} b^k q^{(d-2r)k}&\equiv 0\pmod {[n]}, \end{aligned}$$

where $$0\leqslant m\leqslant n-1$$ and $$dm\equiv -r\pmod {n}$$.

### Proof

It is clear that Lemma 2.2 is true for $n=1$ or $r=0$. We now assume that $n>1$ and $r\ne 0$. By Lemma 2.1 (which is clearly also true for $m=0$) one sees that, for $0\leqslant k\leqslant m$, the *k*th and $(m-k)$th terms on the left-hand side of (2.2) cancel each other modulo $\Phi _n(q)$, i.e.,

$$\begin{aligned}&[2d(m-k)+r]\frac{(aq^r,q^r/a,q^r/b,q^r;q^d)_{m-k}}{(aq^d,q^d/a,bq^d,q^d;q^d)_{m-k}} b^{m-k} q^{(d-2r)(m-k)} \\&\quad \equiv -[2dk+r]\frac{(aq^r,q^r/a,q^r/b,q^r;q^d)_k}{(aq^d,q^d/a,bq^d,q^d;q^d)_k} b^k q^{(d-2r)k}\pmod {\Phi _n(q)}. \end{aligned}$$

This proves that the *q*-congruence (2.2) holds modulo $\Phi _n(q)$.

Moreover, since $dm\equiv -r\pmod {n}$, the expression $(q^r;q^d)_k$ contains a factor of the form $1-q^{\alpha n}$ for $m< k\leqslant n-1$, and is therefore congruent to 0 modulo $\Phi _n(q)$. At the same time the expression $(q^d;q^d)_k$ is relatively prime to $\Phi _n(q)$ for $m< k\leqslant n-1$. Therefore, each summand in (2.3) with *k* in the range $m< k\leqslant n-1$ is congruent to 0 modulo $\Phi _n(q)$. This together with (2.2) modulo $\Phi _n(q)$ establishes the *q*-congruence (2.3) modulo $\Phi _n(q)$.

We are now able to prove (2.2) and (2.3) modulo [*n*]. Let $\zeta \ne 1$ be an *n*th root of unity, not necessarily primitive. Namely, $\zeta$ is a primitive root of unity of degree *s* with $s\mid n$ and $s>1$. Let $c_q(k)$ denote the *k*th term on the left-hand side of (2.3), i.e.,

$$\begin{aligned} c_q(k) =[2dk+r]\frac{(aq^r,q^r/a,q^r/b,q^r;q^d)_k}{(aq^d,q^d/a,bq^d,q^d;q^d)_k}b^k q^{(d-2r)k}. \end{aligned}$$

The *q*-congruences (2.2) and (2.3) modulo $\Phi _n(q)$ with $n\mapsto s$ imply that

$$\begin{aligned} \sum _{k=0}^{m_1}c_\zeta (k)=\sum _{k=0}^{s-1}c_\zeta (k)=0, \end{aligned}$$

where $dm_1\equiv -r\pmod {s}$ and $0\leqslant m_1\leqslant s-1$. We have

2.4 $$\begin{aligned} \lim _{q\rightarrow \zeta }\frac{c_q(\ell s+k)}{c_q(\ell s)} =\frac{c_\zeta (\ell s+k)}{c_\zeta (\ell s)} =\frac{c_\zeta (k)}{[r]}. \end{aligned}$$

It follows that

2.5 $$\begin{aligned} \sum _{k=0}^{n-1}c_\zeta (k)=\sum _{\ell =0}^{n/s-1} \sum _{k=0}^{s-1}c_\zeta (\ell s+k) =\frac{1}{[r]}\sum _{\ell =0}^{n/s-1}c_\zeta (\ell s) \sum _{k=0}^{s-1}c_\zeta (k)=0, \end{aligned}$$

and

$$\begin{aligned} \sum _{k=0}^{m}c_\zeta (k) =\frac{1}{[r]}\sum _{\ell =0}^{(m-m_1)/s-1} c_\zeta (\ell s)\sum _{k=0}^{s-1}c_\zeta (k) +\frac{c_\zeta (m-m_1)}{[r]}\sum _{k=0}^{m_1}c_\zeta (k)=0. \end{aligned}$$

This means that the sums $\sum _{k=0}^{n-1}c_q(k)$ and $\sum _{k=0}^{m}c_q(k)$ are both divisible by the cyclotomic polynomial $\Phi _s(q)$. Since this is true for any divisor $s>1$ of *n*, we deduce that they are divisible by

$$\begin{aligned} \prod _{s\mid n,\, s>1}\Phi _s(q)=[n], \end{aligned}$$

thus establishing the *q*-congruences (2.2) and (2.3). $\square$

We now give the following result, which is a generalization of [13, Theorem 4.2].

### Lemma 2.3

Let *d*, *n*, *r* be integers satisfying $d\geqslant 2$ and $n\geqslant d-r$, such that *d* and *r* are coprime, and $n\equiv -r\pmod {d}$. Let *a*, *b* be indeterminates. Then, modulo $[n](1-aq^{dn-n})(a-q^{dn-n})$,

2.6 $$\begin{aligned}&\sum _{k=0}^{M}[2dk+r]\frac{(aq^r,q^r/a,q^r/b,q^r;q^d)_k}{(aq^d,q^d/a,bq^d,q^d;q^d)_k} b^k q^{(d-2r)k} \nonumber \\&\quad \equiv \frac{(q^{2r}/b;q^d)_{(dn-n-r)/d}}{(bq^d;q^d)_{(dn-n-r)/d}}[dn-n]\left( \frac{b}{q^r}\right) ^{(dn-n-r)/d}, \end{aligned}$$

where $$M=(dn-n-r)/d$$ or $$n-1$$.

### Proof

By the condition in the lemma, we have $r\ne 0$. Recall that Jackson’s ${}_6\phi _5$ summation formula can be written as

2.7 $$\begin{aligned} \sum _{k=0}^N\frac{(1-aq^{2k})(a,b,c,q^{-N};q)_k}{(1-a)(q,aq/b,aq/c,aq^{N+1};q)_k}\biggl (\frac{aq^{N+1}}{bc}\biggr )^k =\frac{(aq,aq/bc;q)_N}{(aq/b,aq/c;q)_N} \end{aligned}$$

(see [2, Appendix (II.21)]). Letting $q\mapsto q^d$, $a=q^r$, $b\mapsto q^r/b$, $c=q^{dn-n+r}$ and $N=(dn-n-r)/d$ in (2.7), we obtain

2.8 $$\begin{aligned}&\sum _{k=0}^{M}\frac{[2dk+r](q^{r-dn+n},q^{r+dn-n},q^r/b,q^r;q^d)_k}{[r](q^{d-dn+n},q^{d+dn-n},bq^d,q^d;q^d)_k} b^k q^{(d-2r)k} \nonumber \\&\quad =\frac{(q^{d+r},bq^{d-dn+n-r};q^d)_{(dn-n-r)/d}}{(bq^d,q^{d-dn+n};q^d)_{(dn-n-r)/d}} \nonumber \\&\quad = \dfrac{(q^{2r}/b;q^d)_{(dn-n-r)/d} [dn-n]}{(bq^d;q^d)_{(dn-n-r)/d} [r]}\left( \frac{b}{q^r}\right) ^{(dn-n-r)/d} . \end{aligned}$$

Namely, when $a=q^{dn-n}$ or $a=q^{n-dn}$ the two sides of (2.6) are equal. Thus, the *q*-congruence (2.6) holds modulo $(1-aq^{dn-n})(a-q^{dn-n})$.

On the other hand, by Lemma 2.2, we see that the left-hand side of (2.6) is congruent to 0 modulo [*n*]. Since $[dn-n]$ is also congruent to 0 modulo [*n*] and $(bq^d;q^d)_{(dn-n-r)/d}$ is coprime with [*n*], we conclude that (2.6) also holds modulo [*n*]. The proof then follows from the fact that $(1-aq^{dn-n})(a-q^{dn-n})$ and [*n*] are coprime polynomials.

Note that the condition $n\geqslant d-r$ means that $(dn-n-r)/d\leqslant n-1$, which is used in the identity (2.8) for $M=n-1$. $\square$

### Lemma 2.4

Let *d*, *n*, *r* be integers satisfying $d\geqslant 2$ and $n\geqslant d-r$, such that *d* and *r* are coprime, and $n\equiv -r\pmod {d}$. Let *a*, *b* be indeterminates. Then

2.9 $$\begin{aligned}&\sum _{k=0}^{M}[2dk+r]\frac{(aq^r,q^r/a,q^r/b,q^r;q^d)_k}{(aq^d,q^d/a,bq^d,q^d;q^d)_k} b^k q^{(d-2r)k} \nonumber \\&\quad \equiv \frac{(q^r,q^{d-r};q^d)_{(dn-n-r)/d}}{(aq^d,q^d/a;q^d)_{(dn-n-r)/d}}[dn-n] \pmod {b-q^{dn-n}}, \end{aligned}$$

where $$M=(dn-n-r)/d$$ or $$n-1$$.

### Proof

Letting $q\mapsto q^d$ and taking $a=q^r$, $b=aq^r$, $c=q^r/a$ and $N=(dn-n-r)/d$ in (2.7), we obtain

$$\begin{aligned}&\sum _{k=0}^{(dn-n-r)/d} \frac{[2dk+r](aq^r,q^r/a,q^{r-dn+n},q^r;q^d)_k}{[r](aq^d,q^d/a,q^{d+dn-n},q^d;q^d)_k}q^{(dn-n+d-2r)k} \\&\qquad =\frac{(q^{d+r},q^{d-r};q^d)_{(dn-n-r)/d}}{(aq^d,q^d/a;q^d)_{(dn-n-r)/d}}. \end{aligned}$$

Namely, when $b=q^{dn-n}$ both sides of (2.9) are equal. This proves the desired *q*-congruence. $\square$

## Proof of Theorems 1.1 and 1.4

With the help of Lemmas 2.3 and 2.4, we can prove the main theorems in this paper now. We need to establish the following parametric generalization of Theorem 1.1.

### Theorem 3.1

Let *d*, *n*, *r* be integers satisfying $d\geqslant 2$, $r\leqslant d-2$, and $n\geqslant d-r$, such that *d* and *r* are coprime, and $n\equiv -r\pmod {d}$. Let *a* be an indeterminate. Then, modulo $[n]\Phi _n(q)(1-aq^{dn-n})(a-q^{dn-n})$,

3.1 $$\begin{aligned}&\sum _{k=0}^{M}[2dk+r]\frac{(aq^r,q^r/a,q^r,q^r;q^d)_k}{(aq^d,q^d/a,q^d,q^d;q^d)_k} q^{(d-2r)k} \nonumber \\&\quad \equiv \frac{(q^{2r};q^d)_{(dn-n-r)/d}}{(q^d;q^d)_{(dn-n-r)/d}}[dn-n]q^{r(n-dn+r)/d}, \end{aligned}$$

where $$M=(dn-n-r)/d$$ or $$n-1$$.

### Proof

It is clear that the polynomials $[n](1-aq^{dn-n})(a-q^{dn-n})$ and $b-q^{dn-n}$ are coprime. By the Chinese remainder theorem for coprime polynomials, we can determine the remainder of the left-hand side of (2.6) modulo $[n](1-aq^{dn-n})(a-q^{dn-n})(b-q^{dn-n})$ from (2.6) and (2.9). To this end, we require the following *q*-congruences:

$$\begin{aligned} \frac{(b-q^{dn-n})(ab-1-a^2+aq^{dn-n})}{(a-b)(1-ab)}&\equiv 1\pmod {(1-aq^{dn-n})(a-q^{dn-n})}, \\ \frac{(1-aq^{dn-n})(a-q^{dn-n})}{(a-b)(1-ab)}&\equiv 1\pmod {b-q^{dn-n}}. \end{aligned}$$

Thus, from (2.6) and (2.9) we deduce that, modulo $[n](1-aq^{dn-n})(a-q^{dn-n})(b-q^{dn-n})$,

3.2 $$\begin{aligned}&\sum _{k=0}^{M}[2dk+r]\frac{(aq^r,q^r/a,q^r/b,q^r;q^d)_k}{(aq^d,q^d/a,bq^d,q^d;q^d)_k} b^k q^{(d-2r)k} \nonumber \\&\quad \equiv \frac{(q^{2r}/b;q^d)_{(dn-n-r)/d}}{(bq^d;q^d)_{(dn-n-r)/d}}\frac{(b-q^{dn-n})(ab-1-a^2+aq^{dn-n})}{(a-b)(1-ab)}\nonumber \\&\qquad \times \, [dn-n]\big (bq^{-r}\big )^{(dn-n-r)/d}\nonumber \\&\qquad \;+\,\frac{(q^r,q^{d-r};q^d)_{(dn-n-r)/d}}{(aq^d,q^d/a;q^d)_{(dn-n-r)/d}}\frac{(1-aq^{dn-n})(a-q^{dn-n})}{(a-b)(1-ab)}[dn-n]. \end{aligned}$$

Note that $1-q^{dn-n}$ has the factor $\Phi _n(q)$ and so do $(q^{2r};q^d)_{(dn-n-r)/d}$ and $(q^{d-r};q^d)_{(dn-n-r)/d}$ since they contain the factors $1-q^{(d-2)n}$ and $1-q^n$, respectively (we need to check that $2r+2n\leqslant dn$, which is guaranteed by the condition $r\leqslant d-2$ and $n\geqslant d-r$ in the theorem). Moreover, the factor $(bq^d;q^d)_{M}$ in the denominator of the left-hand side of (3.2) is coprime with $\Phi _n(q)$ when $b=1$. Thus, letting $b=1$ in (3.2) and observing that

$$\begin{aligned} (1-q^{dn-n})(1+a^2-a-aq^{dn-n})&=(1-a)^2+(1-aq^{dn-n})(a-q^{dn-n}), \end{aligned}$$

we see that the right-hand of (3.2) reduces to

$$\begin{aligned} \frac{(q^{2r};q^d)_{(dn-n-r)/d}}{(q^d;q^d)_{(dn-n-r)/d}}[dn-n]q^{r(n-dn+r)/d} \end{aligned}$$

modulo ${\Phi _n(q)^2(1-aq^{dn-n})(a-q^{dn-n})}$, thus establishing Eq. (3.1) modulo $\Phi _n(q)^2(1-aq^{dn-n})(a-q^{dn-n})$. On the other hand, the *q*-congruences (2.2) ($m=(dn-n-r)/d$ in this case) and (2.3) are also true for $b=1$. That is, the *q*-congruence (3.1) holds modulo [*n*]. The proof then follows from the fact that the least common multiple of $\Phi _n(q)^2(1-aq^{dn-n})(a-q^{dn-n})$ and [*n*] is just $[n]\Phi _n(q)(1-aq^{dn-n})(a-q^{dn-n})$. $\square$

### Proof of Theorem 1.1

Since $(1-q^{dn-n})^2$ contains the factor $\Phi _n(q)^2$ and $(q^d;q^d)_M$ is coprime with $\Phi _n(q)$, letting $a=1$ in (3.1), we are led to

$$\begin{aligned}&\sum _{k=0}^{M}[2dk+r]\frac{(q^r;q^d)_k^4}{(q^d;q^d)_k^4}q^{(d-2r)k}\\&\quad \equiv q^{r(n+r-dn)/d}\dfrac{(q^{2r};q^d)_{(dn-n-r)/d}}{(q^d;q^d)_{(dn-n-r)/d}}[dn-n] \pmod {\Phi _n(q)^4}. \end{aligned}$$

By Lemma 2.2, the above *q*-congruence is true modulo [*n*] and is therefore also true modulo $[n]\Phi _n(q)^3$. Further, if $d=2$ then by the condition in the theorem, one sees that $r<0$ and $-r<(n-r)/2$ and so $(q^{2r};q^2)_{(n-r)/2}$ vanishes. This completes the proof. $\square$

### Proof of Theorem 1.4

Let $d\geqslant 3$ and take $r=1$ and $M=n-1$ in (3.1). Noticing that $(q^2;q^d)_{(dn-n-1)/d}$ is congruent to 0 modulo $\Phi _n(q)$ (we have mentioned this before) and $(q^d;q^d)_{(dn-n-1)/d}$ is coprime with $\Phi _n(q)$, we see that (1.10) holds modulo $\Phi _n(q)^2$. By Lemma 2.2, it also holds modulo [*n*]. This proves the theorem. $\square$

## Concluding Remarks

Using Lemma 2.1, we may also prove the following result similar to Lemma 2.2.

### Lemma 4.1

Let *d*, *n* be positive integers with $\gcd (d,n)=1$. Let *r* be an integer. Then

$$\begin{aligned}&\sum _{k=0}^{m} [2dk+r]\frac{(q^r;q^d)_k^{2d}}{(q^d;q^d)_k^{2d}}q^{d(d-1-r)k} \equiv 0\pmod {[n]}, \\&\sum _{k=0}^{n-1} [2dk+r]\frac{(q^r;q^d)_k^{2d}}{(q^d;q^d)_k^{2d}}q^{d(d-1-r)k} \equiv 0\pmod {[n]}, \end{aligned}$$

where $$0\leqslant m\leqslant n-1$$ and $$dm\equiv -r\pmod {n}$$.

Thus, the *q*-congruence (1.9) (i.e., [10, Theorem 1]) can be further strengthened as follows.

### Theorem 4.2

Let *d*, *n*, *r* be integers satisfying $d\geqslant 3$, $r\leqslant d-2$, and $n\geqslant d-r$, such that *d* and *r* are coprime, and $n\equiv -r\pmod {d}$. Then

4.1 $$\begin{aligned} \sum _{k=0}^{M}[2dk+r] \frac{(q^r;q^d)_k^{2d}}{(q^d;q^d)_k^{2d}}q^{d(d-1-r)k} \equiv 0 \pmod {[n]\Phi _{n}(q)^3}, \end{aligned}$$

where $$M=(dn-n-r)/d$$ or $$n-1$$.

Similarly to the proof of Lemma 2.3, we can also prove the following generalization of [13, Theorem 4.2].

### Theorem 4.3

Let *d* and *n* be positive integers with $n\equiv 1\pmod {d}$. Then, modulo $[n](1-aq^n)(a-q^n)$,

4.2 $$\begin{aligned}&\sum _{k=0}^{m}[2dk+1]\frac{(aq,q/a,q/b,q;q^d)_k}{(aq^d,q^d/a,bq^d,q^d;q^d)_k} b^k q^{(d-2)k}\nonumber \\&\quad \equiv \frac{(b/q)^{(n-1)/d} (q^2/b;q^d)_{(n-1)/d}}{(bq^d;q^d)_{(n-1)/d}}[n], \end{aligned}$$

where $$m=(n-1)/d$$ or $$n-1$$.

Moreover, using the Chinese remainder theorem for coprime polynomials, we may prove that, for such *n* and *d*, modulo $[n](1-aq^n)(a-q^n)(b-q^n)$,

4.3 $$\begin{aligned}&\sum _{k=0}^{m}[2dk+1]\frac{(aq,q/a,q/b,q;q^d)_k}{(aq^d,q^d/a,bq^d,q^d;q^d)_k} b^k q^{(d-2)k} \nonumber \\&\quad \equiv \frac{(b/q)^{(n-1)/d} (q^2/b;q^d)_{(n-1)/d}}{(bq^d;q^d)_{(n-1)/d}}\frac{(b-q^n)(ab-1-a^2+aq^n)}{(a-b)(1-ab)}[n] \nonumber \\&\qquad +\frac{(q,q^{d-1};q^d)_{(n-1)/d}}{(aq^d,q^d/a;q^d)_{(n-1)/d}} \frac{(1-aq^n)(a-q^n)}{(a-b)(1-ab)}[n]. \end{aligned}$$

For $d=1$, the *q*-congruence (4.2) is just an identity (with a telescoping truncated sum). For $d=2$, letting $b=1$ in (4.3), we get (1.6), which further reduces to (1.3) when $m=(n-1)/2$ and *a* tends to 1. This is what the first author obtained in [6]. For $d\geqslant 3$, we are unable to deduce, by first letting $b=1$ and then taking $a\rightarrow 1$, a concrete interesting *q*-congruence modulo $\Phi _n(q)^4$ from (4.3) (because there appears to be no simple formula for the limit).

We conclude our paper with the following two conjectural *q*-supercongruences related to Theorem 3.1. (In the following conjectures, we actually consider the difference of the sums in (3.1), determined by the two endpoints $M=n-1$, and $M=(dn-n-r)/d$. We observed that this difference has a factor, as specified in Conjectures 4.4 and 4.5.) In the case $d=2$ and $r=1$ they coincide and were recently confirmed by the first author [6, Theorem 6.1].

### Conjecture 4.4

Let *d*, *n* be positive integers and *r* a (possibly negative) integer with $dn-n\geqslant r\geqslant d-n$, such that *d* and *r* are coprime, and $n\equiv -r\pmod d$. Then, modulo $[n]\Phi _n(q)(1-aq^{dn-n})(a-q^{dn-n})(b-q^{dn-n})$,

$$\begin{aligned} \sum _{k=(dn-n-r+d)/d}^{n-1}[2dk+r] \frac{(a q^r,q^r/a,q^r/b,q^r;q^d)_k}{(a q^d,q^d/a,bq^d,q^d;q^d)_k}b^kq^{(d-2r)k} \equiv 0. \end{aligned}$$

### Conjecture 4.5

Let *d*, *n* be positive integers and *r* a (possibly negative) integer with $n\geqslant r\geqslant n-dn+d$, such that *d* and *r* are coprime, and $n\equiv r\pmod d$. Then, modulo $[n]\Phi _n(q)(1-aq^n)(a-q^n)(b-q^n)$,

$$\begin{aligned} \sum _{k=(n-r+d)/d}^{n-1}[2dk+r] \frac{(a q^r,q^r/a,q^r/b,q^r;q^d)_k}{(a q^d,q^d/a,bq^d,q^d;q^d)_k}b^kq^{(d-2r)k} \equiv 0. \end{aligned}$$
